# Novel Colloidal MoS_2_ Quantum Dot Heterojunctions on Silicon Platforms for Multifunctional Optoelectronic Devices

**DOI:** 10.1038/srep29016

**Published:** 2016-06-30

**Authors:** Subhrajit Mukherjee, Rishi Maiti, Ajit K. Katiyar, Soumen Das, Samit K. Ray

**Affiliations:** 1 Advanced Technology Development Centre, Indian Institute of Technology, Kharagpur- 721302, India; 2 Department of Physics, Indian Institute of Technology, Kharagpur- 721302, India; 3 School of Medical Science and Technology, Indian Institute of Technology, Kharagpur-721302, India

## Abstract

Silicon compatible wafer scale MoS_2_ heterojunctions are reported for the first time using colloidal quantum dots. Size dependent direct band gap emission of MoS_2_ dots are presented at room temperature. The temporal stability and decay dynamics of excited charge carriers in MoS_2_ quantum dots have been studied using time correlated single photon counting spectroscopy technique. Fabricated n-MoS_2_/p-Si 0D/3D heterojunctions exhibiting excellent rectification behavior have been studied for light emission in the forward bias and photodetection in the reverse bias. The electroluminescences with white light emission spectra in the range of 450–800 nm are found to be stable in the temperature range of 10–350 K. Size dependent spectral responsivity and detectivity of the heterojunction devices have been studied. The peak responsivity and detectivity of the fabricated heterojunction detector are estimated to be ~0.85 A/W and ~8 × 10^11^ Jones, respectively at an applied bias of −2 V for MoS_2_ QDs of 2 nm mean diameter. The above values are found to be superior to the reported results on large area photodetector devices fabricated using two dimensional materials.

Two dimensional (2-D) transition metal dichalcogenides (TMDC) materials, exhibiting room temperate direct bandgap emission in monolayer form, have been found to be promising for future electronic and optoelectronic devices^1^,^2^,^3^,^4^,^5^,^6^. MoS_2_, a member of TMDC family, has attracted much attention due to its tunable optical band gap, efficient multiple carrier generation, high chemical stability, earth abundance and nontoxicity^7^,^8^,^9^,^10^. Nominally undoped mono-to-few layers thick MoS_2_ behaves as an n-type direct band gap semiconductor^11^,^12^. Due to the difficulty in making reliable MoS_2_ homojunctions, van-der-Waal’s heterostructures are being studied recently with MoS_2_ in combination with other p-type 2D materials^13^,^14^,^15^,^16^,^17^,^18^. However, till date all the devices have been reported using monolayer or few layers MoS_2_ nanosheets, with very small active area requiring complex nanolithography process, which are unsuitable for Si CMOS compatible optoelectronic applications. In recent years, solution processed colloidal graphene/carbon quantum dots based optoelectronic devices have received considerable attention because of low cost manufacturing process, integration compatibility to various substrates including the flexible ones and size-dependent tunable device properties^19^,^20^,^21^,^22^,^23^,^24^,^25^,^26^.

In this study, we report the novel vertical MoS_2_/Si heterojunctions consisting of colloidal n-type MoS_2_ quantum dots (QDs) integrated with p-type Si for multifunctional optoelectronic devices. Strong absorption and size dependent bandgap of MoS_2_ QD have been exploited to fabricate heterojunction photodetector and light emitting devices (LED). The electroluminescence (EL) characteristics of fabricated n-MoS_2_/p-Si vertical heterostructures exhibit white light emissions in the spectral range ~450 nm to ~800 nm on forward bias condition. The fabricated 0D/3D vertical heterostructure with short carrier diffusion path has resulted in higher photoresponsivity and detectivity, due to excellent light absorbing property of MoS_2_ QDs and charge separation phenomena due to the built-in electric field at the abrupt heterojunction. This study reveals the potential of colloidal MoS_2_ based novel hybrid devices on silicon platform for future large area, ultra-dense nanophotonic integrated circuits.

## Results and Discussion

**Figure 1:**
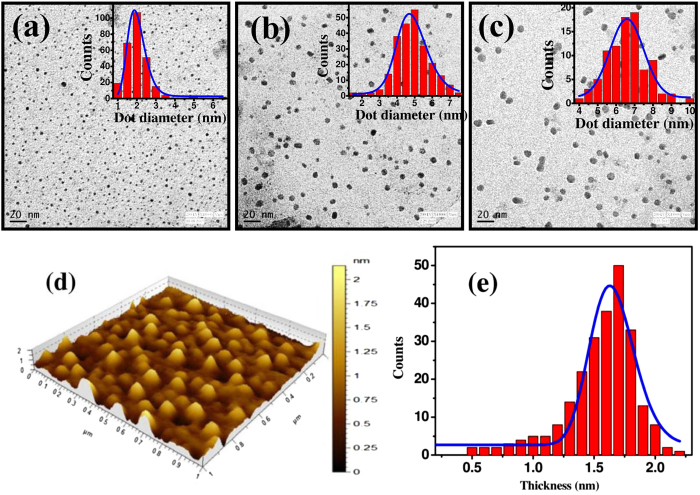
TEM micrographs of synthesized MoS_2_ quantum dots using centrifugation speeds of (**a**) 15000, (**b**) 14000 and (**c**) 12000 rpm. Corresponding inset exhibits the size distribution of quantum dots with fitted curves (blue line) presented for a different centrifugation speed. (**d**) Typical atomic force 3D topographic micrograph of MoS_2_ quantum dots synthesized using a centrifugation speed of 15000 rpm and coated onto a Si substrate. Scale represents height variation along z-axis. (**e**) Histogram obtained from the corresponding image, representing the thickness distribution of QDs on a Si substrate.

Variable sized MoS_2_ quantum dots have been synthesized by solvent assisted controlled sonication-centrifugation process. Figure 1(a–c) present the TEM micrographs of the QDs grown with a centrifugation speed of 15000, 14000 and 12000 rpm, respectively. All micrographs reveal the formation of well dispersed and nearly circular quantum dots. Corresponding inset histograms exhibits the size distribution of MoS_2_ QDs synthesized with a centrifugation speed of 15000, 14000 and 12000 rpm, respectively. The corresponding average size of QDs is extracted to be ~2, ~5 and ~7 nm, respectively. The morphology and height of as-prepared MoS_2_ QDs have been studied by atomic force microscopy. Typical 3-D surface topographic image with a corresponding histogram of the height distribution of spin coated MoS_2_ QDs synthesized using a centrifugation speed 15000 rpm are depicted in Fig. 1(d,e). Figure 1(d) indicates almost uniform and narrow size distribution of MoS_2_ QDs with an average thickness of ~1.8 nm. The average thickness corresponds to the 2–3 layers of MoS_2_.

**Figure 2:**
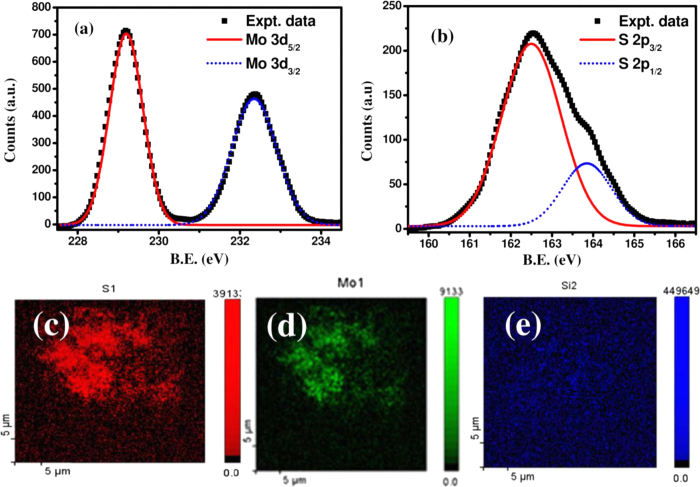
High-resolution XPS spectra showing the binding energy of (**a**) Mo 3d and (**b**) S 2p electrons. Scanning auger electron spectroscopy (AES) images of MoS_2_ films deposited on Si substrates mapping the elemental distribution for (**c**) Mo, (**d**) S and (**e**) Si recorded in the same region.

The chemical bonding and composition of as-synthesized MoS_2_ QDs have been studied by X-ray photoelectron spectroscopy (XPS) using Al–Kα radiation of energy 1486.6 eV. High-resolution core-level XPS spectra showing the binding energy of Mo 3d and S 2p electrons are presented in Fig. 2(a,b), respectively. Two distinct peaks for Mo 3d electrons observed at 229.2 and 232.3 eV are attributed to 3d_5/2_ and 3d_3/2_ chemical states, respectively. The spectrum for S 2p electrons is deconvoluted into two well resolved Gaussian peaks located at 162.5 and 163.8 eV, owing to S 2p_3/2_ and S 2p_1/2_ states, respectively. The observed peak positions of spin-orbit coupled Mo 3d and S 2p electrons are in excellent agreement with those reported in literature^27^,^28^, confirming the synthesis of pure 2-H phase MoS_2_. The atomic percentage ratio of Mo and S in MoS_2_ QDs has been estimated by considering the atomic sensitivity factor and area under the S 2p and Mo 3d peaks with Shirley background correction. The atomic ratio of S to Mo is extracted to be 2.12, indicating the formation of a slight S-rich MoS_2_. A broad energy XPS survey spectrum is presented in Figure S1 (ESI copy), showing a weak but distinct signal of Cl 2p electrons, in addition to O and C as surface contaminants. The presence of Cl as an impurity nominally induces an n-type doping in MoS_2_ samples^13^,^29^. Figure 2(c–e), respectively, present the Auger electron spectroscopic (AES) elemental mapping images of S, Mo and Si atoms for MoS_2_ QDs deposited on Si. The individual elemental map reveals the homogeneous distribution of two different elements (Mo and S), which are present only at the specific areas of MoS_2_ layers on Si surface.

**Figure 3:**
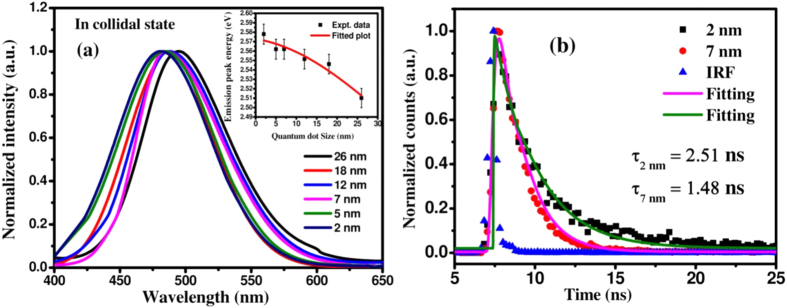
(**a**) Typical PL characteristics of the synthesized QDs in solution exhibiting size tunable emission. Inset of figure (**a**) shows the variation of peak position as a function of dot diameter. (**b**) Photoluminescence decay spectra of 2 nm and 7 nm dia MoS_2_ QDs along with the instrument response function of TCSPS set up. Solid lines are fit to the experimental data using eqn. (1).

Figure 3(a) exhibits typical photoluminescence spectra of colloidal MoS_2_ QDs in DMF solvent for different diameters revealing the emission peak in the range of ~480–500 nm for the size varying from ~2 to 26 nm. The shift of PL peak energy as a function of QDs size is presented in the inset of Fig. 3(a). The emission peak energy is gradually shifted to a higher energy with the reduction of QDs size, in well agreement with the quantum confinement effect. Quantum confinement effect has a notable influence on the band gap of nanostructures, when their size is comparable or smaller than the excitonic Bohr radius. The excitonic Bohr radius of MoS_2_ has been estimated to be ~23 nm. [see in ESI copy]. The photocarrier lifetime of generated excitons in MoS_2_ QDs have been investigated by employing the time-correlated single photon counting spectroscopy (TCSPS) of colloidal MoS_2_ QDs using a 380 nm pulsed diode laser excitation source. Figure 3(b) exhibits the typical PL decay spectra of 2 nm and 7 nm dia QDs, along with the instrument response function (IRF). To compare the photogenerated carrier lifetime upon excitation, the experimental data have been fitted, using Marquardt-Levenberg algorithm. All the spectra can be fitted (solid lines) reasonably with a single exponential decay equation, convoluted with a Gaussian IRF described by the equation^30^:


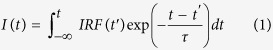


where τ is the photon-excited carrier life time and the χ^2^ value is found to be ~0.98 in the numerical fitting parameter. The well fitted single exponential decay time indicates the existence of only single radiative recombination channel of excitons with negligible contribution from trap states in MoS_2_ QDs. From the fitting of PL decay curve, the estimated carrier life time is found to be ~1.48 ns and ~2.51 ns, for 7 nm and 2 nm dia QDs, respectively. The larger size QDs exhibits a shorter life time than those of the smaller ones. Therefore with decreasing diameter, the radiative lifetime becomes progressively longer. This is a direct experimental evidence of the temporal stability of photoexcited charge carriers in MoS_2_ QDs. The observed exciton lifetime is found to be much superior compared to the complex multi-exponential carrier lifetime of ~10 ps observed for layered MoS_2_ structure^31^,^32^. The presence of lower defect density is also consistent with a much higher PL quantum yield of QDs than that of monolayered MoS_2_ (~10^−3^) sheet.

**Figure 4:**
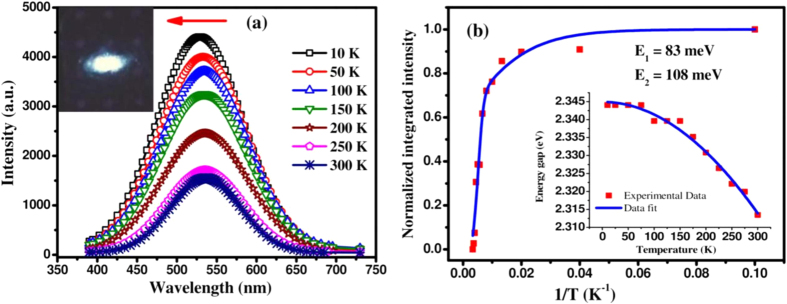
(**a**) Typical temperature dependent photoluminescence spectra of MoS_2_ QDs showing the enhancement of emission intensity at lower temperatures. Optical image of the photoluminescence captured for the MoS_2_/Si heterojunction. (**b**) Temperature dependent integrated PL intensity of the synthesized QDs of size ~2 nm. The solid line shows the best fitted curve for the experimental data with two activation energies. Inset shows the temperature dependent energy gap for ~2 nm size QDs sample, where the solid line represents the fitting of the experimental data using Varshni’s relation.

To explore the origin of PL emission, the temperature dependent PL measurements of ~2 nm dia MoS_2_ QDs have been carried out in the temperature range 10 K to 300 K and the results are depicted in Fig. 4(a). With decreasing temperature, the observed PL peak (~2.31 eV) intensity is enhanced and a slight but distinguishable and progressive blue shift is noticed. Inset is a photograph of white colour PL emission from MoS_2_ QDs/Si, which instigates to make an LED device. The temperature dependent integrated PL intensity for the ~2 nm dia MoS_2_ QDs is presented in Fig. 4(b). The PL intensity as a function of inverse temperature has been fitted using the relation^33^,


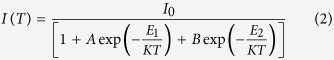


where I(T) is the integrated PL intensity at a particular temperature T, ‘A’ and ‘B’ are constants indicating the ratio of nonradiative to radiative recombination probabilities. The activation energy E_1_ and E_2_ implies the non-radiative recombination for thermal quenching at a higher temperature. From the best fitting of the data using above equation, thermal activation energies E_1_ and E_2_ for PL quenching are found to be 3.24 meV and 59.95 meV, respectively. This indicates that the solution processed MoS_2_ QDs have a few surface states, due to the local deviation from stoichiometry. This is corroborated by XPS and AES results showing S-rich MoS_2_ on the surface of quantum dots. Therefore, the observed PL quenching at higher temperature with an activation energy of ~3 meV may be associated with the detrapping of localized charge carriers due to the slight band offset between the core and the surface states of the QDs. The higher activation energy of ~60 meV is related to the dissociation of photo-generated excitons through nonradiative recombination centers. The inset of Fig. 4(b) shows the PL peak shift as a function of temperature. The variation of peak position with temperature has been fitted by Varshni’s equation^34^, describing the band gap reduction with temperature for semiconductors.


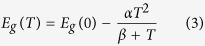


where E_g_(0) is the direct band gap energy at 0 K, α is constant, and β is the Einstein temperature for MoS_2_. The values of α and β are found to be 0.0036 and 10233 K, respectively. The well fitted curve indicates the direct bandedge transition in quantum confined MoS_2_ dots.

**Figure 5:**
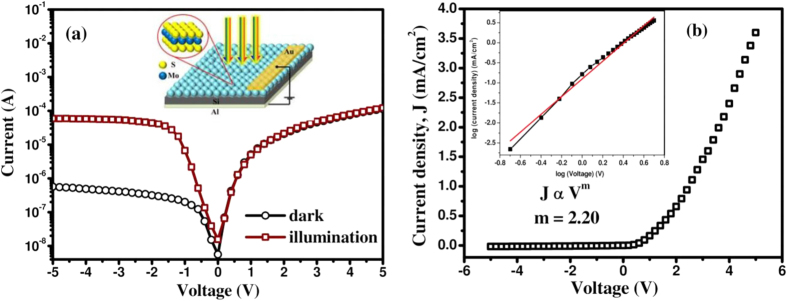
(**a**) Current-voltage characteristics of fabricated heterojunction, using ~2 nm MoS_2_ quantum dots on Si, under dark and illumination condition. The inset shows the schematic structure of the heterojunction device. (**b**) J–V characteristics of fabricated p-n heterojunctions under dark condition, at room temperature. The inset shows the logarithmic J–V curve for the heterojunction fitted with a power law J ∝V^m^ corresponding to the trap-charge-limited current transport.

To demonstrate the potential of Si CMOS compatible devices, p-n heterostructures using MoS_2_ quantum dots of variable diameter have been fabricated on p-Si substrates. The XPS survey scan has shown the incorporation of Cl as an impurity, which results in an n-type doping of MoS_2_. The efficiency of n-MoS_2_/p-Si heterojunction for applications in light emitting devices (LED) and photodetectors has been studied. Typical current-voltage (I–V) characteristic of n-MoS_2_/p-Si heterojunction under dark and illumination conditions is depicted in Fig. 5(a), with the inset showing the device structure. The asymmetric nature of the dark I–V characteristics clearly indicates the formation of a good quality p-n junction at the interfaces with a fairly low dark current (~4.10 × 10^−7^ A at −3 V). Upon illumination (514 nm) a photo-to-dark current ratio of ~132 is achieved. The current density (J) versus voltage (V) characteristics of the fabricated heterojunction is shown in Fig. 5(b), which has been fitted with Richardson-Schottky diode equation. The estimated diode ideality factor of the fabricated heterojunction is found to be ~9. The relatively large ideality factor originates due to the existence of high density interfacial defects states in solution processed QDs layer over p-Si resulting in thermal generation-recombination current. The log-log plot of J–V characteristic for the MoS_2_/Si vertical heterojunction is best fitted with the equation J ∝V^m^, which is shown in the inset of Fig. 5(b). The slope m ~2.2, clearly reveals that the charge transport across the p-n junction could be explained by a model described by the charge trapping at the interfacial defect sites, which limit the output current.

**Figure 6:**
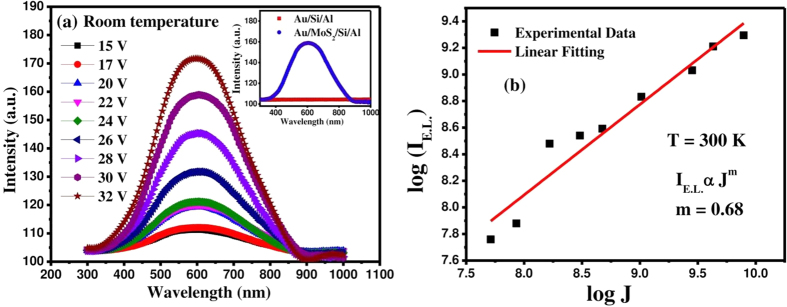
(**a**) Bias dependent electroluminescence spectra of MoS_2_/Si heterojunction recorded at room-temperature. EL characteristics of the control device (without MoS_2_ quantum dots and MoS_2_/Si heterojunction are shown in the inset for comparison. (**b**) The integrated EL intensity fitted against the current density with a power law.

The electroluminescence (EL) characteristics of the fabricated MoS_2_/Si heterojunction for applications in LED devices have been studied in forward bias condition using Al-doped ZnO (AZO) as the transparent top electrode. A bluish-white light emission could be observed by the naked eye, when sufficient current is injected across the device at room temperature. The EL spectra of MoS_2_-Si vertical heterostructure are presented under different applied bias and current injection (~0.22–1.98 mA/cm^2^) in Fig. 6(a). For a comparative study, we have tested the control device without MoS_2_ QDs, under an applied bias of 30 V. A comparison of the EL emission intensity of control (Au/Si/Al) and the fabricated heterojunction (Au/MoS_2_/Si/Al) devices is depicted in the inset of Fig. 6(a). The absence of EL emission from the control device unambiguously establishes the importance of p-Si/n-MoS_2_ heterojunction for achieving the visible-light LED. A broad band spectrum from 450 to 800 nm covering the full visible range indicates the white light emission from MoS_2_/Si heterojunction. A detectable EL emission is observed above a threshold carrier injection around ~0.2 mA/cm^2^ and increases continuously with the increase of current across the junction (up to ~2 mA/cm^2^), as shown in Fig. 6(b). The applied voltage for fairly detectable emission is rather high in agreement with the reported value (~12 V) in the literature^35^. A higher threshold bias compared to the conventional semiconductor device is owing to the high resistance of nominally undoped MoS_2_ QDs leading to a low injection current density. The emission peak centered at around ~580 nm is in close agreement with the PL spectra. The integrated EL intensity (I_EL_) versus injected current density characteristics is found to obey a power law (I_EL_ ∝ J^n^), where the exponent “n” accounts for the influence of defects in the light emission characteristics. The exponent value extracted through the linear fitting of the log−log plot of I_EL_ versus J in Fig. 6(b) is found to be ~0.68 for 300 K, is comparable to the reported values in literature^36^. This sub-linear dependence of integrated EL intensity on injected current is attributed to the presence of non-radiative recombination centers at the interface of Si and MoS_2_, which also lead to a high dark current density. The stability of the fabricated LEDs has been tested over a wide range of temperatures from 10 to 350 K and the results are presented in Figure S2 (ESI copy). Only a slight decrease in EL intensity is observed with increasing the temperature from 10 to 350 K, indicating the potential of heterojunction LED operating at an elevated temperature. The results indicate the potential of lithography-free, solution processed MoS_2_ QDs for the fabrication of large-area white light emitting devices on existing Si platforms.

**Figure 7:**
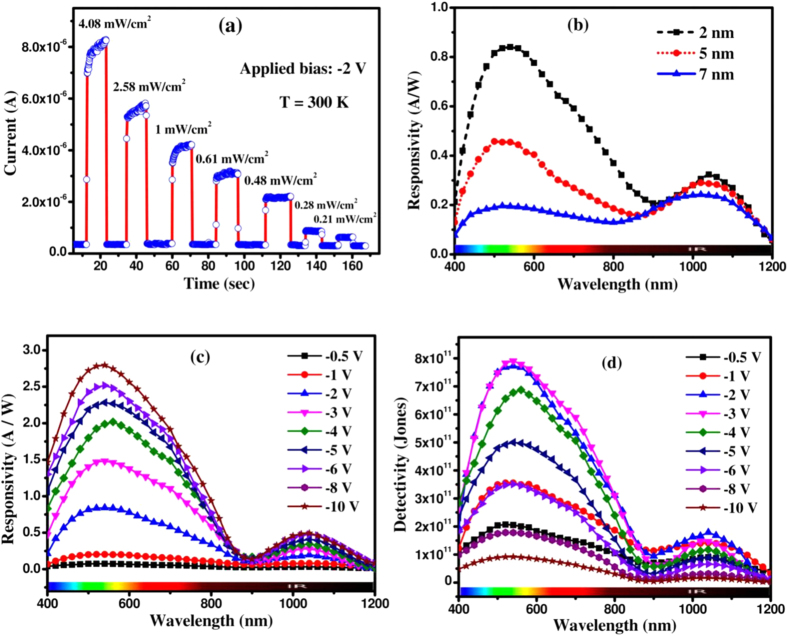
(**a**) Optical modulation characteristics of the fabricated MoS_2_/Si heterojunction device (using ~2 nm QDs) upon pulsed illumination (514 nm) for variable input intensity at room temperature. (**b**) Size dependent spectral responsivity of the fabricated heterojunction for different MoS_2_ QD size, recorded at a bias of −2 V and optical power of 50 μW. (**c**) Bias dependent response of the device fabricated using QDs of average size ~2 nm. (**d**) Detectivity in the broadband spectral range of fabricated photodetectors of QD size ~2 nm for different bias.

The MoS_2_/Si heterojunction has also been used to demonstrate the photodetection in reverse bias condition. Figure 7(a) exhibits the temporal time response of the heterojunction detector upon pulsed optical excitation (λ = 514 nm) with varying illumination intensity at an applied bias of −2 V at room temperature. The device current increases sharply and stabilizes in a high conductivity state (ON state) upon illumination and switches back quickly to a lower conductivity state (OFF state) in dark condition. Responsivity (R), detectivity (D) and external quantum efficiency (EQE) are the important parameters to characterize the performance of a photodetector. The spectral responsivity of a photodetector is directly proportional to the internal gain showing the response of the device towards the incident radiation and can be expressed by^37^:


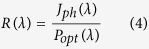


where, J_ph_(λ) is the photocurrent density and P_opt_(λ) is the optical power density for a specific wavelength. Figure 7(b) presents the spectral responsivity of the fabricated heterojunction devices using different sized MoS_2_ QDs recorded at −2 V bias. The broad spectral response from visible to near-IR consists of two prominent peaks centered around 550 nm and 1030 nm. The peak centered at ~550 nm is attributed to the photo-absorption in MoS_2_ QDs and the one at ~1030 nm is associated with the intrinsic band-edge transition of Si. It is interesting to note that the responsivity due to QDs increases significantly with the reduction of QD size, as shown in the Fig. 7(b). On the other hand, responsivity due to absorption in Si remains same. The results are in corroboration with the transient PL study (Fig. 3(b)), where the smaller QDs exhibited higher carrier lifetime resulting in a higher detector gain. The higher surface recombination rate results in a lower responsivity for larger sized QDs. The observed peak responsivity for ~2 nm dia MoS_2_ QDs is significantly higher than the graphene/Si vertical heterostructure (~200 mA/W)^38^,^39^ as well as commercial Si (~600 mA/W)^40^,^41^ photodetectors.

**Figure 8:**
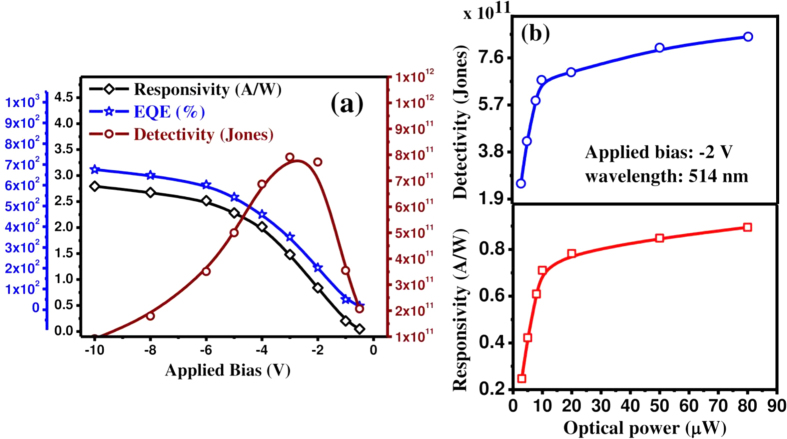
(**a**) Combined plots of peak responsivity, EQE and detectivity of MoS_2_ QD (~2 nm)/Si heterojunction photodetector as a function of applied bias. (**b**) Responsivity and detectivity behavior as a function of illuminated optical power for a fixed wavelength (514 nm) and an applied bias (−2 V).

**Figure 9:**
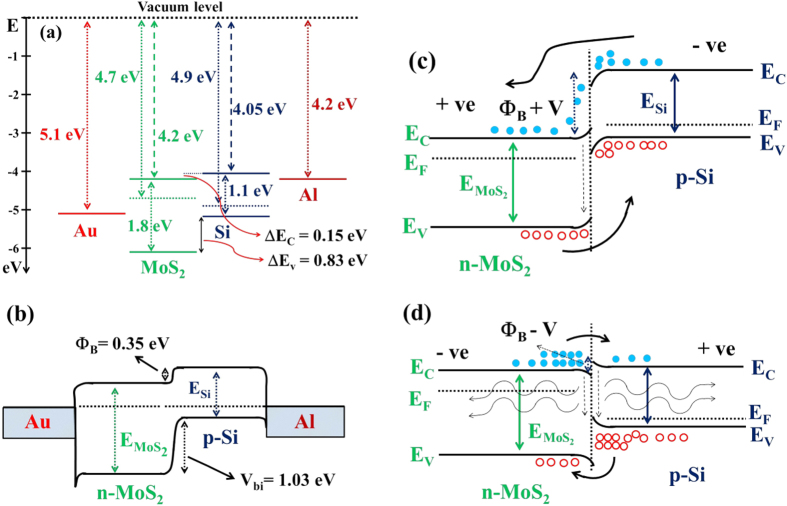
(**a**) Estimated band edge alignment of Au, n-MoS_2_, p-Si and Al to realize the band diagram of type-II heterojunction. Energy band diagram of the fabricated Au/n-MoS_2_/p-Si/Al heterojunction under (**b**) equilibrium (zero bias), (**c**) reverse bias and (**d**) forward bias conditions.

The influence of applied electric field on photocurrent collection efficiently of fabricated device is shown in Fig. 7(c). The peak responsivity centered at ~550 nm due to MoS_2_ QDs is enhanced significantly with increasing applied electric field, as compared to that of Si. The EQE spectra as a function wavelength for different QDs diameter is also studied and presented in Figure S3(a) (ESI copy), which also enhanced with reduction of QD sizes. The figure of merit of a photodetector is determined in terms of specific detectivity (D^*^) and noise equivalent power (NEP), which can be expressed as^37^,


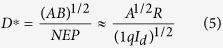


where D* is measured in cm.Hz^1/2^.W^−1^ (Jones), A the effective detector area in cm^2^, R the responsivity, I_d_ the dark current and q is the electronic charge. We assume that the shot noise from dark current is the dominant source of the total noise in comparison to Johnson and flicker noise, which are mainly attributed to thermal fluctuations. Figure 7(d) exhibits the specific detectivity of the heterojunction using 2 nm dia MoS_2_ QDs in broad wavelength range for different bias. The peak detectivity of the fabricated p-n heterojunction diode is estimated to be ~7 × 10^11 ^cm-Hz^1/2^.W^−1^ (or jones) for −2 V bias. The detectivity has also been estimated for variable size MoS_2_ QDs over the spectral range of 400–1200 nm and is depicted in Figure S3(b) (ESI copy). The detectivity is found to be enhanced by 4-fold as the QDs diameter reduces from ~7 nm to ~2 nm. Peak spectral responsivity, external quantum efficiency (EQE) and detectivity are plotted together against applied reverse bias for the device with 2 nm dia MoS_2_ QD and the results are presented in Fig. 8(a). As the applied voltage across the p-n junction increases, the responsivity as well as EQE increase sublinearly, but a noticeably different phenomenon is observed for the detectivity. As the bias increases from 0 V, the detectivity increases up to the peak value of ~8 × 10^11^ Jones and thereafter reduces with the further increase of applied bias. Therefore the detector performance has been found to be optimum at a bias of −2 V for the fabricated MoS_2_/Si heterostructures. We also examined the responsivity and detectivity of fabricated devices for variable incident optical power and results are depicted in Fig. 8(b). Both the responsivity and detectivity increase linearly for the low illumination power, but exhibit a sub-linear dependence at higher power levels. The increase of illumination intensity leads to the enhanced electron–hole pair generation rate, resulting in a higher photocurrent. However, a sub-linear behavior of the responsivity indicates that the optical response is also affected by the localized trapping and recombination of charge carriers near the band edges. The responsivity and detectivity of the present MoS_2_ QD/Si heterostructure device were compared with those reported in the literature for similar type photodetectors and are presented in Table 1. The mechanisms of the operation of QD-based photodetectors and LEDs can be explained by the help of energy band diagrams, as shown in Fig. 9. The conduction and valence band edges of Si and MoS_2_ and the work function edges of Au and Al are aligned in proper energy scale, as shown in Fig. 9(a), resulting in a type-II heterojunction band alignment. Figure 9(b) exhibits the band diagram of the Au/MoS_2_/Si/Al heterojunction under an equilibrium condition (zero bias). A large barrier potential is created at the Si/QDs interface, when the junction is formed between p-type Si with a work function (Φ_Si_) of 4.9 eV and n-type MoS_2_ QDs (Φ_MoS2_ ~ 4.7 eV). In quantum dot based device, the photo generated charge carriers are transported by the hopping mechanism via interparticle barriers. Upon irradiation with energy larger than the band gap, excitons are generated at the p-n junction. Owing to edge defects of small sized QDs and the potential arising at the interface, the excitons are likely to be dissociated at the interface, and the electrons and holes start drifting towards the opposite electrodes in the presence of electric field, resulting in the photocurrent. With increase in reverse bias, the corresponding electric field across the depletion region is gradually enhanced, resulting in an expansion of the barrier potential as well as the depletion region, as shown in Fig. 9(c). Then enhanced transport of the photoexcited carriers leads to the higher responsivity. Under the application of a forward bias, charge carriers are injected across the junction, resulting in favorable electron-hole recombination. This leads to the visible light emission through radiative recombination of charge carriers injected into the Si/MoS_2_ heterojunction, which could be explained using the proposed energy band diagram under a forward bias, as shown in Fig. 9(d). At the forward bias condition, the barrier potential at the interface becomes lower which is ideal for the efficient injection of electrons from MoS_2_ and holes from p-Si. The increase of bias leads to the enhanced charge carrier transport rate and the resultant light emission intensity. The results indicate the potential of MoS_2_ QD/Si heterojunction for application in high performance multifunctional optoelectronic devices.

## Conclusions

We have demonstrated the multifunctional optoelectronic devices using vertical heterojunctions of colloidal MoS_2_ quantum dots integrated on a Si platform. XPS spectra have shown the formation of nearly stoichiometric 2H phase MoS_2_ with nominal n-type doping due to the presence of Cl. The size of the quantum dots using solvent assisted sonication-centrifugation process could be varied from 2–26 nm exhibiting size tunable emission. Time resolved PL study shows the existence of single radiative recombination centres of MoS_2_ QDs with superior carrier life time (1.5–2.5 ns), as compared to MoS_2_ flakes (~10 ps). The fabricated 0D/3D heterostructures exhibit rectifying characteristics with a relatively high junction ideality factor of ~9.0. Bias dependent electroluminescence in the broad spectral range (450–800 nm) of the heterojunction in the forward bias condition shows its efficacy for the LED applications. The spectral responsivity due to MoS_2_ quantum dots is found to be significantly enhanced with the reduction of size, exhibiting a peak responsivity of 0.85 A/W and a peak detectivity of ~8 × 10^11^ Jones at −2 V for ~2 nm dia quantum dots. The reported values are much higher than that of commercial Si homojunction, graphene/Si heterojunction and colloidal graphene/carbon dot based devices. The study shows the potential of colloidal n-MoS_2_ QDs for Si compatible large area multifunctional optoelectronic devices using 0D/3D heterojunctions.

## Methods

MoS_2_ quantum dots were synthesized using the solvent assisted sono-chemical exfoliation process reported previously^41^,^42^,^43^,^44^. Since the present investigations have been focused on the fabrications of vertical Si/MoS_2_ p-n heterojunction, a continuous film over clean Si surface is essential. But, it has been observed that the high boiling (~155 °C) dimethylformamide (DMF) leads to the self-aggregation of QDs resulting in a sporadic film. To transfer the QDs from DMF to a suitable solvent for ease of device fabrication, the exfoliation process was modified. After the prolong sonication, the DMF dispersions of MoS_2_ was vigorously stirred for 2 days to get the homogeneous dots size distribution confirmed by microscopic measurement. Subsequently, MoS_2_ QDs precipitates of different size were obtained through gradual centrifugation after sonication. All the precipitates were collected separately and a mild heat treatment (at ~60 °C) was performed under the argon (Ar) atmosphere to evaporate the excessive solvent. Finally, all the dried precipitations were redispersed into ethanol, spin coated onto the H-passivated Si surface and dried at room temperature to make a continuous film. The B-doped Si substrates (p-type) with a resistivity of 0.77 Ω-cm were used. Device fabrication was completed by the thermal evaporation of Au (~70 nm) as top electrode and Al (~80 nm) at the backside of Si for bottom electrode, with base pressure of ~1 × 10^−6^ torr. However, for LED fabrication, approximately 20 nm of transparent and conducting Al-doped ZnO (AZO) film, was deposited by pulsed laser deposition (PLD) system using a KrF excimer laser (λ = 248 nm, τ = 25 ns) under an optimized condition (base pressure ~5 × 10^−6^ Torr, substrate temperature ~300 °C, energy density of ~2 J/cm^2^ and repetition rate ~5 Hz). The deposited AZO film had a resistivity of ~10^−3^ Ω.cm and an average transmittance of ~92% in the visible wavelength. Surface morphology of the MoS_2_ QDs was studied using field emission scanning electron microscopy (FESEM), transmission electron microscopy (TEM) and atomic force microscopy (AFM). The chemical composition and valence states were studied using X-ray photoelectron spectroscopy (XPS) system. The optical absorption spectra of the MoS_2_ QDs were recorded using an ultraviolet-visible spectrophotometer. Temperature dependent photoluminescence and electroluminescence spectra were recorded using TRIAX-320 monochromator and Hamamatsu R928 PMT detector combination and a He-Cd laser as an excitation source (325 nm). The current-voltage (I–V) characteristics of the fabricated p-n junction and photo-current response of the device was measured using a set-up consisting of a monochromator, calibrated broadband light source and a Keithley semiconductor parameter analyzer (Model 4200-SCS). The temporal response of the PD was recorded using an Ar laser (514 nm) source with a mechanical chopper.

## Additional Information

**How to cite this article**: Mukherjee, S. *et al*. Novel Colloidal MoS_2_ Quantum Dot Heterojunctions on Silicon Platforms for Multifunctional Optoelectronic Devices. *Sci. Rep.* **6**, 29016; doi: 10.1038/srep29016 (2016).

## Supplementary Material

Supplementary Information

## Figures and Tables

**Table 1:** Comparison of the performance of MoS_2_ and heterojunction based photodetectors.

Device	Responsivity (A/W)	Detectivity (Jones)	References
Mechanically exfoliated MoS_2_ flakes	0.1155	10^10^	45
CVD grown MoS_2_ film	0.001	Not reported	47
RF sputtered MoS_2_ film	1.8	2 × 10^9^	48
Si based photodetector	0.4 A/W*0.60.59	1.55 × 10^10^	*commercial Si- detector, Newport, Model 818-BB-214041
Si-MoS_2_heterojunction	70.04	10^10^	18
Graphene–silicon heterojunction	0.2250.210.13	2.1 × 10^8^	50
Graphene–MoS_2_ junction	0.221.26~10	4.2 × 10^10^	51
MoS_2_ QD/Si heterostructure	2.8	0.8 × 10^12^	**This work**

## References

[b1] Huang X., Zeng Z. Y. & Zhang H. Metal Dichalcogenide Nanosheets: Preparation, Properties and Applications. Chem. Soc. Rev. 42, 1934–1946 (2013). 23344899 10.1039/c2cs35387c

[b2] Wang Q. H., Kalantar-Zadeh K., Kis A., Coleman J. N. & Strano M. S. Electronics and optoelectronics of two-dimensional transition metal dichalcogenides. Nat. Nanotech. 7, 699–712 (2012). 10.1038/nnano.2012.193 23132225

[b3] Jariwala D., Sangwan V. K., Lauhon L. J., Marks T. J. & Hersam M. C. Emerging Device Applications for Semiconducting Two-Dimensional Transition Metal Dichalcogenides. ACS Nano. 8, 1102–1120 (2014). 24476095 10.1021/nn500064s

[b4] Nicolosi V., Chhowalla M., Kanatzidis M. G., Strano M. S. & Coleman J. N. Liquid Exfoliation of Layered Materials. Science 340, 1226419 (2013).

[b5] Chhowalla M., Liu Z. & Zhang H. Two-Dimensional Transition Metal Dichalcogenide (TMD) Nanosheets. Chem. Soc. Rev. 44, 2584–2586 (2015). 25882213 10.1039/c5cs90037a

[b6] Huang X., Tan C., Yin Z. & Zhang H. 25th Anniversary Article: Hybrid Nanostructures Based on Two-Dimensional Nanomaterials. Adv. Mater. 26, 2185–2204 (2014). 24615947 10.1002/adma.201304964

[b7] Li H., Wu J., Yin Z. & Zhang H. Preparation and Applications of Mechanically Exfoliated Single-Layer and Multilayer MoS_2_ and WSe_2_ Nanosheets. Acc. Chem. Res. 47, 1067–1075 (2014). 24697842 10.1021/ar4002312

[b8] Mak K. F., Lee C., Hone J., Shan J. & Heinz T. F. Atomically thin MoS_2_: a new direct-gap semiconductor. Phys. Rev. Lett. 105, 136805 (2010). 21230799 10.1103/PhysRevLett.105.136805

[b9] Splendiani A. . Emerging Photoluminescence in Monolayer MoS_2_. Nano Lett. 10, 1271–1275 (2010). 20229981 10.1021/nl903868w

[b10] Lopez-Sanchez O., Lembke D., Kayci M., Radenovic A. & Kis A. Ultrasensitive photodetectors based on monolayer MoS_2_. Nat. Nanotech. 8, 497–501 (2013). 10.1038/nnano.2013.100 23748194

[b11] Radisavljevic B., Radenovic A., Brivio J., Giacometti V. & Kis A. Single-Layer MoS_2_ Transistors. Nat. Nanotech. 6, 147–150 (2011). 10.1038/nnano.2010.279 21278752

[b12] Yin Z. . Single-Layer MoS_2_ Phototransistors. ACS Nano. 6, 74–80 (2012). 22165908 10.1021/nn2024557

[b13] Gong Y. . Vertical and in-plane heterostructures from WS_2_/MoS_2_ monolayers. Nat. Mater. 13, 1135–1142 (2014). 25262094 10.1038/nmat4091

[b14] Ceballos F., Bellus M. Z., Chiu H. Y. & Zhao H. Ultrafast Charge Separation and Indirect Exciton Formation in a MoS_2_/MoSe_2_ van der Waals Heterostructure. ACS Nano. 8, 12717–12724 (2014). 25402669 10.1021/nn505736z

[b15] Deng Y. . Black Phosphorus Monolayer MoS_2_ van der Waals Heterojunction p-n Diode. ACS Nano. 8, 8292–8299 (2014). 25019534 10.1021/nn5027388

[b16] Tsai M. L. . Monolayer MoS_2_ Heterojunction Solar Cells. ACS Nano. 8, 8317–8322 (2014). 25046764 10.1021/nn502776h

[b17] Lopez-Sanchez O. . Light Generation and Harvesting in a van der Waals Heterostructure. ACS Nano. 8, 3042–3048 (2014). 24601517 10.1021/nn500480u PMC3971963

[b18] Li Y., Xu C. Y., Wang J. Y. & Zhen L. Photodiode-like behavior and excellent photoresponse of vertical Si/monolayer MoS_2_ heterostructures. Sci Rep. 26, 7186 (2014). 25424301 10.1038/srep07186 PMC4244624

[b19] Li X., Rui M., Song J., Shen Z. & Zeng H. Carbon and Graphene Quantum Dots for Optoelectronic and Energy Devices: A Review. Adv. Funct. Mater. 25, 4929–4947 (2015).

[b20] Zhang X. . Color-Switchable Electroluminescence of Carbon Dot Light-Emitting Diodes. ACS Nano. 7, 11234–11241 (2013). 24246067 10.1021/nn405017q

[b21] Jin Z., Owour P., Lei S. & Ge L. Graphene, graphene quantum dots and their applications in optoelectronics. Current Opinion in Colloid & Interface Science. 20, 439–453 (2015).

[b22] Konstantatos G. . Hybrid graphene–quantum dot phototransistors with ultrahigh gain. Nat. nanotech. 7, 363–368 (2012). 10.1038/nnano.2012.60 22562036

[b23] Li L. S. & Yan X. Colloidal Graphene Quantum Dots. J. Phys. Chem. Lett. 1, 2572–2576 (2010).

[b24] Zhang Q. . Solution-Processed Graphene Quantum Dot Deep-UV Photodetectors. ACS Nano. 9, 1561–1570 (2015). 25625624 10.1021/acsnano.5b00437

[b25] Park H. . Large Scale Synthesis and Light Emitting Fibers of Tailor-Made Graphene Quantum Dots. Sci. Rep. 5, 14163 (2015). 26383257 10.1038/srep14163 PMC4585659

[b26] Lin H. . Colloidal synthesis of MoS_2_ quantum dots: size-dependent tunable photoluminescence and bioimaging. New J. Chem. 39, 8492–8497 (2015).

[b27] Kibsgaard J., Chen Z., Reinecke B. N. & Jaramillo T. F. Engineering the surface structure of MoS_2_ to preferentially expose active edge sites for electrocatalysis. Nat. Mater. 11, 963–969 (2012). 23042413 10.1038/nmat3439

[b28] Jin Z., Shin S., Kwon D. H., Han S. J. & Min Y. S. Novel chemical route for atomic layer deposition of MoS_2_ thin film on SiO_2_/Si substrate. Nanoscale 6, 14453–14458 (2014). 25340905 10.1039/c4nr04816d

[b29] Sundaram R. S. . Electroluminescence in Single Layer MoS_2_. Nano Lett. 13, 1416–1421 (2013). 23514373 10.1021/nl400516a

[b30] Yuan L. & Huang L. Exciton dynamics and annihilation in WS_2_ 2D semiconductors. Nanoscale. 7, 7402–7408 (2015). 25826397 10.1039/c5nr00383k

[b31] Korn T., Heydrich S., Hirmer M., Schmutzler J. & Schüller C. Low-temperature photocarrier dynamics in monolayer MoS_2_. Appl. Phys. Lett. 99, 102109 (2011).

[b32] Lagarde D. . Carrier and Polarization Dynamics in Monolayer MoS_2_. Phy. Rev. Lett. 112, 047401 (2014). 10.1103/PhysRevLett.112.047401 24580489

[b33] Palm J., Gan F., Zheng B., Michel J. & Kimerling L. C. Electroluminescence of erbium-doped silicon. Phy. Rev. B. 54, 17603 (1996). 10.1103/physrevb.54.17603 9985886

[b34] Varshni Y. P. Temperature dependence of the energy gap in semiconductors. Physica. 34, 149–154 (1967).

[b35] Yin Z. . Preparation of MoS_2_–MoO_3_ Hybrid Nanomaterials for Light-Emitting Diodes. Angew. Chem. Int. Ed. 126, 12768–12737 (2014). 10.1002/anie.201402935 25047022

[b36] Katiyar A. K., Sinha A. K., Manna S. & Ray S. K. Fabrication of Si/ZnS Radial Nanowire Heterojunction Arrays for White Light Emitting Devices on Si Substrates. ACS Appl. Mater. Interfaces. 6, 15007–15014 (2014). 25137439 10.1021/am5028605

[b37] Das K., Mukherjee S., Manna S., Ray S. K. & Raychaudhuri A. K. Single Si nanowire (diameter ≤ 100 nm) based polarization sensitive near-infrared photodetector with ultra-high responsivity. Nanoscale 6, 11232–11239 (2014). 25126742 10.1039/c4nr03170a

[b38] Maiti R., Manna S., Midya A. & Ray S. K. Broadband Photoresponse and Rectification of Novel Graphene Oxide/n-Si Heterojunctions. Optics express 21, 26034–26043 (2013). 24216828 10.1364/OE.21.026034

[b39] Wang X., Cheng Z., Xu K., Tsang H. K. & Xu J. B. High-responsivity graphene/silicon-heterostructure waveguide photodetectors. Nat. Photonics. 7, 888–891 (2013).

[b40] Zheng J. P., Jiao K. L., Shen W. P., Anderson W. A. & Kwok H. S. Highly sensitive photodetector using porous silicon. Appl. Phys. Lett. 61, 459–461 (1992).

[b41] Ahmad M., Rasool K., Rafiq M. A. & Hasan M. M. Enhanced and persistent photoconductivity in vertical silicon nanowires and ZnS nanoparticles hybrid devices. Appl. Phys. Lett. 101, 223103 (2012).

[b42] Gopalakrishnan D., Damien D. & Shaijumon M. M. MoS_2_ Quantum Dot-Interspersed Exfoliated MoS_2_ Nanosheets. ACS Nano, 8, 5297–5303 (2014). 24773177 10.1021/nn501479e

[b43] Štengl V. & Henychab J. Strongly luminescent monolayered MoS_2_ prepared by effective ultrasound exfoliation. Nanoscale 5, 3387–3394 (2013). 23467444 10.1039/c3nr00192j

[b44] Mukherjee S., Maiti R., Midya A., Das S. & Ray S. K. Tunable Direct Bandgap Optical Transitions in MoS_2_ Nanocrystals for Photonic Devices. ACS Photonics 2, 760–768 (2015).

[b45] Zhang X. . A Facile and Universal Top-Down Method for Preparation of Monodisperse Transition-Metal Dichalcogenide Nanodots. Angew. Chem. Int. Ed. 54, 5425–5428 (2015). 10.1002/anie.201501071 25760801

[b46] Woong C. . High‐detectivity multilayer MoS_2_ phototransistors with spectral response from ultraviolet to infrared. Adv. Mater. 24, 5832–5836 (2012). 22903762 10.1002/adma.201201909

[b47] Kufer D. . G. Hybrid 2D–0D MoS_2_–PbS quantum dot photodetectors. Adv. Mater. 27, 176–180 (2015). 25400160 10.1002/adma.201402471

[b48] Néstor P. L. . CVD-grown monolayered MoS_2_ as an effective photosensor operating at low-voltage. 2D Materials 1, 011004 (2014).

[b49] Ling Z. P. . Large-scale two-dimensional MoS_2_ photodetectors by magnetron sputtering. Optics express. 23, 13580–13586 (2015). 26074606 10.1364/OE.23.013580

[b50] Midya A., Ghorai A., Mukherjee S., Maiti R. & Ray S. K. Hydrothermal growth of few layer 2H-MoS_2_ for heterojunction photodetector and visible light induced photocatalytic applications. J. Mater. Chem. A. 4, 4534 (2016).

[b51] An X., Liu F., Jung Y. J. & Kar S. Tunable graphene–silicon heterojunctions for ultrasensitive photodetection. Nano letters 13, 909–916 (2013). 23350824 10.1021/nl303682j

[b52] Yu W. J. . Highly efficient gate-tunable photocurrent generation in vertical heterostructures of layered materials. Nat. nanotech. 8, 952–958 (2013). 10.1038/nnano.2013.219 PMC4249654 24162001

[b53] Vabbina P. . Highly Sensitive Wide Bandwidth Photodetector Based on Internal Photoemission in CVD Grown p-Type MoS_2_/Graphene Schottky Junction. ACS appl. mater. & inter. 7, 15206–15213 (2015). 10.1021/acsami.5b00887 26148017

[b54] Xu H. . High Responsivity and Gate Tunable Graphene‐MoS_2_ Hybrid Phototransistor. Small 10, 2300–2306 (2014). 24664627 10.1002/smll.201303670

