# Supplementary material for: Novel Colloidal MoS2 Quantum Dot Heterojunctions on Silicon Platforms for Multifunctional Optoelectronic Devices
Source: Sci Rep. 2016 Jun 30;6:29016. doi: 10.1038/srep29016 (PMC4928078; doi:10.1038/srep29016)
Supplement: Supplementary Information [file srep29016-s1.pdf]

Electronic Supplementary Information (ESI)

Novel Colloidal MoS_2 Quantum Dot Heterojunctions on Silicon Platforms for Multifunctional Optoelectronic Devices

S. Mukherjee[1], *R. Maiti*[2], *A. Katiyar*[2], *S. Das*[3], *S. K. Ray*[2,#]

[1]*Advanced Technology Development Centre*, [2]*Department of Physics*, [3]*School of Medical Science & Technology, IIT Kharagpur, Kharagpur*

#corresponding author: physkr@phy.iitkgp.ernet

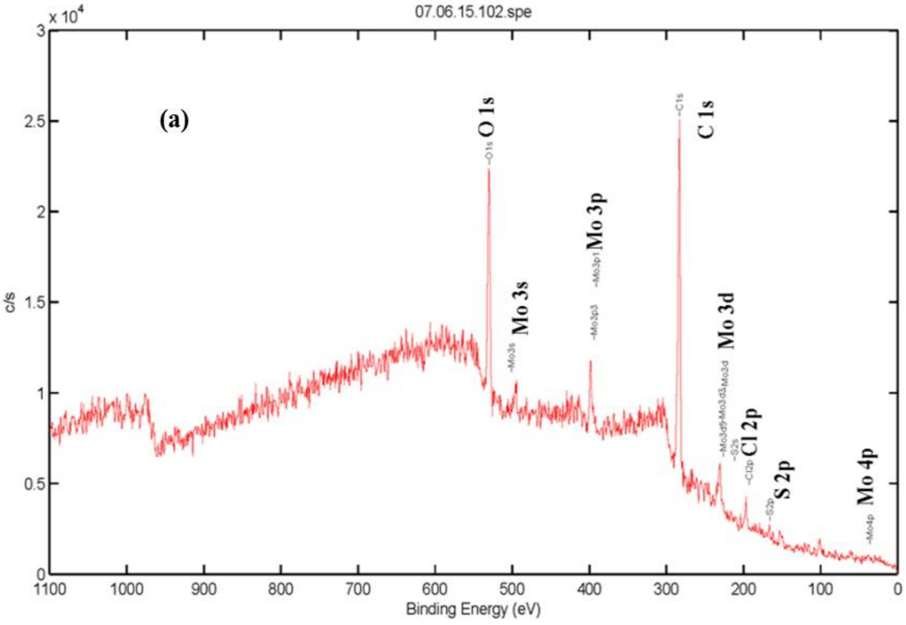

Fig. S1: Broad energy XPS survey spectrum of MoS_2 deposited on Si showing a weak but distinct signal of Cl 2p electrons. The presence of Cl as an impurity indicated an n-type doping in MoS_2 samples.

The excitonic Bohr radius of MoS_2 has been estimated using the following equation:

$$R_B = \frac{\hbar^2 \varepsilon}{e^2}\left[\frac{1}{m_e^*} + \frac{1}{m_h^*}\right] \quad \text{............ (S.1a)}$$

Where, m_e* and m_h* are the effective masses of electrons and holes, respectively, $\hbar$ is the reduced Planck's constant, e is the electronic charge and ε is the relative permittivity of MoS_2. The effective masses and the dielectric permittivity have been considering as $m_h^* = 0.41m_o$ and

$m_e^* = 0.48m_o$, as m_o is the rest mass of the electrons and ~11, respectively. Using eq S.1, the excitonic Bohr radius has been found as ~23 nm.

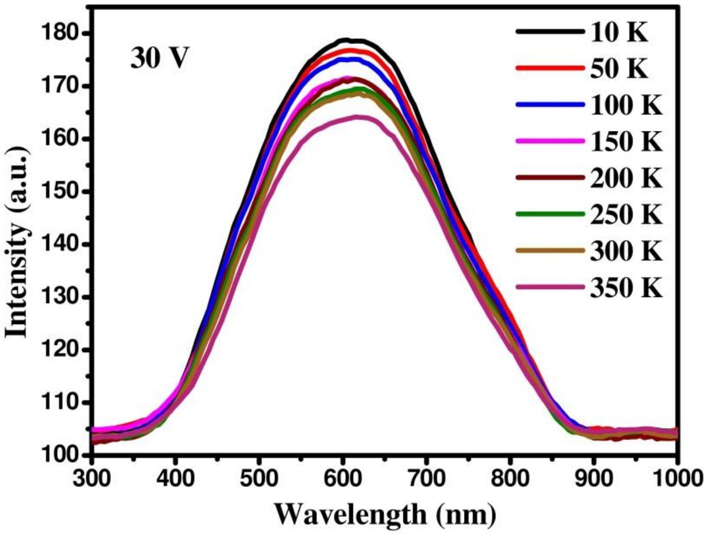

Fig. S2: (a) Temperature dependent electroluminescence (EL) spectra of Si/MoS_2 p-n heterojunction, recorded at 30 V applied bias.

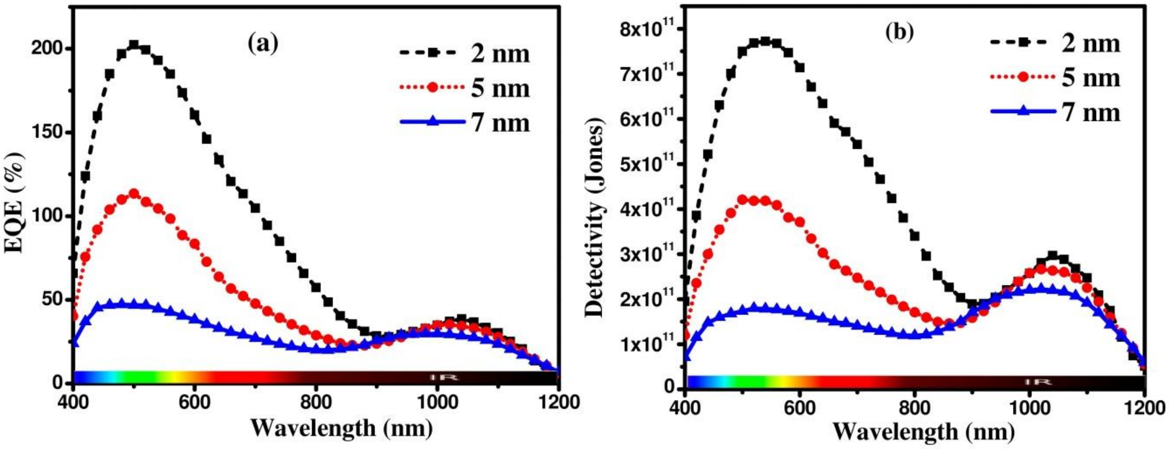

Fig. S3: (a) Spectral dependent external quantum efficiency (EQE) and (b) specific detectivity of different size MoS_2 quantum dot based devices exhibits PDs performances have been strongly dependent on the QDs size. All the spectra recorded at -2 V applied bias. The detectivity and the EQE both have been found ~4-fold improves with the reduction of QDs size from ~7 nm to ~2 nm.
